# Nrf2/Bach1 signaling axis: A promising multifaceted therapeutic strategy for Alzheimer's disease

**DOI:** 10.1016/j.neurot.2025.e00586

**Published:** 2025-04-07

**Authors:** Priyanka Soni, Sudarshana M. Sharma, Andrew A. Pieper, Bindu D. Paul, Bobby Thomas

**Affiliations:** a Darby Children's Research Institute, Medical University of South Carolina, Charleston, SC, USA; b Department of Pediatrics, Medical University of South Carolina, Charleston, SC, USA; c Department of Biochemistry and Molecular Biology and Hollings Cancer Center, Medical University of South Carolina, Charleston, SC, USA; d Department of Psychiatry, Case Western Reserve University, Cleveland, OH, USA; e Brain Health Medicines Center, Harrington Discovery Institute, University Hospitals Cleveland Medical Center, Cleveland, OH, USA; f Geriatric Psychiatry, GRECC, Louis Stokes Cleveland VA Medical Center, Cleveland, OH, USA; g Institute for Transformative Molecular Medicine, School of Medicine, Case Western Reserve University, Cleveland, OH, USA; h Department of Neurosciences, School of Medicine, Case Western Reserve University, Cleveland, OH, USA; i Department of Pathology, School of Medicine, Case Western Reserve University, Cleveland, OH, USA; j Department of Pharmacology and Molecular Sciences, Johns Hopkins University School of Medicine, Baltimore, MD, USA; k The Solomon H. Snyder Department of Neuroscience, Johns Hopkins University School of Medicine, Baltimore, MD, USA; l Department of Psychiatry and Behavioral Sciences, Johns Hopkins University School of Medicine, Baltimore, MD, USA; m Lieber Institute for Brain Development, Baltimore, MD, USA; n Department of Neuroscience, Medical University of South Carolina, Charleston, SC, USA; o Department of Drug Discovery, Medical University of South Carolina, Charleston, SC, USA

**Keywords:** Nrf2, Bach1, Alzheimer's disease, Oxidative stress, Neuroinflammation

## Abstract

Alzheimer's disease (AD) is the most prevalent form of dementia, which continues to elude effective treatment despite decades of research and numerous clinical trials. While existing therapeutic strategies have primarily targeted neuropathological hallmarks such as amyloid plaques and tau tangles, they have failed to halt disease progression, leaving patients with limited options. This persistent failure reveals a critical gap in our understanding of AD and calls for a fresh perspective - one that goes beyond the traditional targets and dives deeper into the fundamental cellular processes that drive neurodegeneration. Recent advances in molecular biology underscore the significance of nuclear factor E2-related factor 2 (Nrf2), often termed the "guardian of redox homeostasis," in the pathophysiology of AD. Nrf2 orchestrates cellular responses to oxidative stress and neuroinflammation - two interlinked pathological features of AD. In the brains of AD patients, Nrf2 activity is diminished, weakening the brain's ability to counteract oxidative damage. Additionally, the BTB and CNC homology 1 (Bach1) protein, a transcriptional repressor of Nrf2, has emerged as a potential therapeutic target. Here, we review the current landscape of clinical trials in AD and identify the limitations of the conventional approaches. We then explore the prospects of a novel approach that combines Nrf2 activation with Bach1 inhibition to achieve a multipronged defense against oxidative stress, neuroinflammation, and other molecular culprits driving AD. This innovative strategy holds promise for synergistically modulating multiple neuroprotective pathways to advance AD treatment.

## Introduction

Alzheimer's disease (AD) remains one of the most complex and devastating neurodegenerative disorders, accounting for approximately 60–80 ​% of dementia cases globally, characterized by the progressive degeneration of neurons, primarily in the cortical and hippocampal regions. As per the World Alzheimer Report, over 55 million people around the world have AD or similar conditions, which is further expected to rise to 82 million by 2030 and reach 138 million by 2050 [1,2]. In the United States alone, more than 6.7 million individuals aged 65 and older are currently living with AD (prevalence rate of 10.7 ​%). This number is projected to more than double to 13 million by 2050 [1,2]. The pathological hallmarks of AD include the formation of senile plaques composed of aggregated insoluble amyloid beta (Aβ) peptides [[3], [4], [5]] and neurofibrillary tangles (NFTs), which comprise hyperphosphorylated tau, a microtubule-associated protein [6,7]. While significant advances have been made in understanding the molecular underpinnings of AD, oxidative stress has emerged as a pivotal driver of disease progression, contributing to neuronal dysfunction and degeneration [8,9]. The brain, with its high metabolic activity and significant oxygen consumption, is particularly susceptible to oxidative damage, which exacerbates the pathological features of AD [10].

**Fig. 1:**
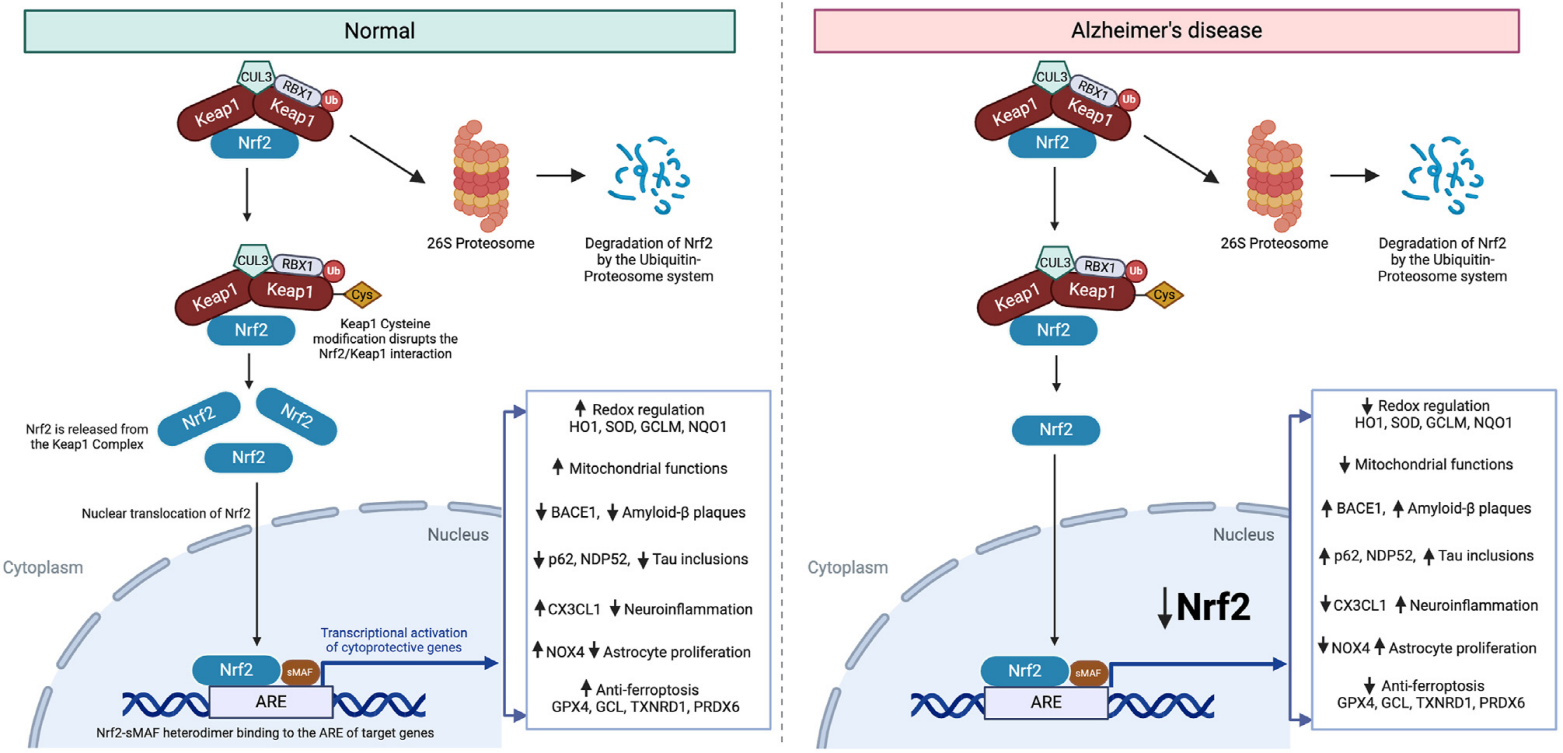
**Nrf2 signaling in Alzheimer's Disease.** An overview of Nrf2 signaling pathway in controls and AD brain. In normal cells, Nrf2 is tightly regulated by KEAP1 (Kelch-like ECH-associated protein 1), which forms a complex with Nrf2 and facilitates its ubiquitination via the E3 ubiquitin ligase complex (CUL3-RBX1), targeting it for degradation by the 26S proteasome. Modifications to KEAP1's cysteine residues disrupt the Nrf2-KEAP1 interaction, allowing Nrf2 to accumulate, translocate into the nucleus, and bind to antioxidant response elements (ARE) as a heterodimer with small MAF proteins. This nuclear Nrf2 binding induces the transcription of genes involved in redox regulation, mitochondrial functions, anti-inflammatory responses, and anti-ferroptosis mechanisms, which promote cellular resilience. In Alzheimer's disease, however, this regulatory mechanism is impaired. Nrf2 is unable to effectively dissociate from KEAP1, leading to its decreased nuclear translocation and reduced activation of cytoprotective genes. This results in a downregulation of antioxidant and anti-inflammatory responses, contributing to AD pathology. AD-related changes include increased amyloid-β plaque formation (via elevated BACE1), tau protein aggregation, neuroinflammation, impaired mitochondrial function, and a compromised anti-ferroptosis response. Created with BioRender.com.

One of the primary defense mechanisms against oxidative stress is the nuclear factor erythroid 2-related factor 2 (Nrf2) pathway, which regulates the expression of genes involved in antioxidant responses, detoxification, mitochondrial metabolism, neuroinflammation, proteostasis, and cellular repair [[11], [12], [13]] (Fig. 1). Under normal, unstressed conditions, Nrf2 is a transient and unstable protein, primarily regulated by Kelch-like ECH-associated protein 1 (KEAP1), a component of the KEAP1-CUL3-RBX1 E3 ubiquitin ligase that facilitates the ubiquitination and subsequent proteasomal degradation of Nrf2 [14]. In response to oxidative stress or treatment with Nrf2 activators, KEAP1 undergoes a conformational change either by electrophilic-mediated covalent modification of KEAP1 cysteine residues or by direct displacement from protein-protein interaction inhibitors of the KEAP1:Nrf2 complex, which prevents the binding and degradation of newly synthesized Nrf2, thus evading proteasomal degradation. This results in Nrf2 stabilization and subsequent translocation into the nucleus, dimerizing with small Maf (musculoaponeurotic fibrosarcoma) proteins [12,15]. The Nrf2-Maf heterodimer then binds to antioxidant response elements (AREs) in the promoter regions of target genes, activating their transcription (Fig. 1). This cascade results in the upregulation of various cytoprotective proteins, including the antioxidant enzymes such as superoxide dismutase (SOD), catalase, and heme oxygenase-1 (HO-1), as well as detoxifying enzymes that facilitate the clearance of harmful metabolites [12].

Recent studies suggest that Nrf2 signaling is downregulated in the AD brain, which may contribute to an inadequate defense against the heightened oxidative stress observed in the disease [16,17]. This impaired response exacerbates oxidative damage and hinders the clearance of toxic proteins such as Aβ and tau, further driving disease pathology [18]. As a result, the Nrf2 pathway has garnered attention as a promising therapeutic target for AD, with the potential to modulate multiple pathogenic processes, including oxidative stress, inflammation, and proteostasis [19]. In addition to activating Nrf2, inhibiting BTB and CNC homology 1 (Bach1), a transcriptional repressor that competes with Nrf2 for ARE binding, has emerged as a novel therapeutic approach. Inhibiting Bach1 can further enhance Nrf2 signaling, boosting the brain's resilience to oxidative damage and promoting neuronal survival. This review explores the current understanding of Nrf2 dysfunction in AD and discusses the therapeutic potential of concurrently modulating the Nrf2 and Bach1 pathways to tackle the complex nature of AD pathology.

## Nrf2 signaling pathway in Alzheimer's disease

Accumulating evidence indicates a dysregulation of Nrf2-mediated pathways in AD. For example, astrocytic Nrf2 expression is significantly reduced by 50 ​% in the frontal cortex of AD patients compared to non-demented control subjects [20]. Concomitantly, the number of astrocytic branches in the frontal cortex decreased by 60 ​%, and their branch length was reduced by 14 ​% compared to controls. A similar reduction in Nrf2+ astrocytes was observed in the 3 ​× ​Tg-AD mouse model at 10 months of age, where the percentage of Nrf2+ astrocytes dropped from 79 ​% in wild-type mice to 41 ​% in 3 ​× ​Tg-AD mice. Additionally, the relative number and branch length of astrocytes in the cortex of 3 ​× ​Tg-AD mice showed reductions of 20 ​% and 19 ​%, respectively, compared to wild-type mice [20]. Notably, a decrease of Nrf2 has also been associated with mitochondrial dysfunction in astrocytes, as indicated by increased mitochondrial fragmentation (from 20 ​% to 70 ​% in Nrf2-silenced cells), smaller and ruptured mitochondria, and elevated 3-nitrotyrosine (3-NT) levels. These mitochondrial abnormalities contribute to the accumulation of amyloid-β plaques, tau inclusions, neuroinflammation, and cognitive deficits in AD models (Fig. 1). Importantly, evidence suggests that Nrf2 activation may help preserve mitochondrial integrity and reduce oxidative and inflammatory stress, emphasizing its therapeutic potential in mitigating AD pathogenesis [20].

In AD postmortem brains, Nrf2 has been found primarily in the cytoplasm, with significantly reduced nuclear localization compared to age-matched healthy controls [21]. The reduction in nuclear Nrf2 has been linked to increased Aβ production [22], and Nrf2 deficiency in APP^V717I^ and TauP^301L^ mice exacerbated amyloid deposition, tau pathology, oxidative stress, neuroinflammation, and cognitive deficits [23,24]. Similarly, Tang et al., observed a reduced expression of Nrf2 with elevated NADPH oxidase 4 (NOX4) expression in astrocytes from the frontal cortex of both AD patients and 3 ​× ​Tg-AD mice [20]. The downregulation of Nrf2 in astrocytes significantly inhibited the expression of crucial Nrf2 targets, with HO-1 and glutathione peroxidase 4 (GPX4) reduced by 27 ​% and 44 ​%, respectively. Conversely, Nrf2 silencing led to a compensatory increase in cystine transporter (xCT) expression by 20 ​% and NOX4 by 21 ​% in astrocytes, reflecting the role of Nrf2 in regulating cellular redox balance [20]. Notably, Nrf2 signaling may be differentially regulated at multiple levels. For example, lipid peroxidation exerts a dual effect on the Nrf2 pathway. While on the one hand, lipid peroxidation products, such as 4-hydroxynonenal (4-HNE), can modify KEAP1 cysteines and thereby activate Nrf2 [12], on the other hand, excessive lipid peroxidation also inhibits Nrf2-regulated antioxidant enzymes, including glutamate-cysteine ligase (GCL), thioredoxin reductase 1 (TXNRD1), and peroxiredoxin 1 (PRDX6), disrupting redox homeostasis and promoting ferroptosis in AD [7,12,25].

In contrast, activation of Nrf2 has been shown to confer neuroprotection in various disease models. For example, in AD astrocytes derived from pluripotent stem cells, pharmacological activation of Nrf2 reduced amyloid-β secretion and inflammatory cytokine expression [26]. Furthermore, in an AD mouse model (App^NLGF^), Nrf2 activation in glial cells helped suppress pro-inflammatory responses and inhibited phagocytic activation, thereby reducing astrocyte proliferation, mitigating neuronal damage around amyloid plaques, increasing glutathione levels, and improving cognitive function [27,28]. Furthermore, Nrf2 activation also minimizes the transition of microglia from a resting to an active state and decreases the proliferation of reactive astrocytes, providing neuroprotective benefits in AD [27,28]. Thus, an increase in Nrf2 protein levels and Nrf2 activity, which activate the downstream neuroprotective Nrf2 target genes, are essential for therapeutic effects in AD.

On the other hand, excessive activation of the Nrf2 pathway causes reductive stress, which can prove deleterious. For example, reductive stress caused by hyper-activation of the antioxidant pathway leads to an increased ratio of the reduced to oxidized NAD(P)H and glutathione forms and may contribute to AD onset [29]. Reductive stress also compromises signaling pathways and protein folding in the endoplasmic reticulum (ER), which may result in the aggregation of misfolded proteins and impaired neurogenesis [30]. Most studies on reductive stress were performed in cancer cell lines. A recent observation pointed to the damaging effect of genetically or pharmacologically hyperactivated Nrf2-driven programs when combined with Complex I inhibition in cancer cells that rely on mitochondrial respiration, not glycolysis [31]. A build-up of NADH inside mitochondria and simultaneous Complex I inhibition will result in mitochondrial failure, as in hypoxia or with stoichiometric antioxidants induced by over-nutrition [32]. Although interesting, the extent to which these observations apply to mechanistic aspects of disease onset and progression in AD is unknown. Notably, a recently published 10-year follow-up study in a population of healthy individuals carrying one copy of the *ApoE 4* gene showed that mild reductive stress resulted in oxidative stress in the study population [33].

Beyond regulating the antioxidant response, Nrf2 also influences AD pathology through other mechanisms [22]. For instance, Nrf2 negatively regulates beta-site amyloid precursor protein cleaving enzyme 1 (BACE1), a key enzyme in amyloid-β production [7,[34], [35], [36]]. Levels of BACE1 are elevated in the brains of AD patients, indicating that dysregulation of BACE1 expression is involved in AD pathogenesis (Fig. 1) [37]. It has been shown that Nrf2 binds to the antioxidant response elements (AREs) in the BACE1 promoter to suppress BACE1 transcription [22]. Activation of Nrf2 reduced BACE1-mediated amyloid-beta generation and alleviated cognitive deficits in AD models [22]. Interestingly, this regulation of BACE1 by Nrf2 appears to be independent of reactive oxygen species (ROS), highlighting a novel mechanism of amyloid-β regulation beyond conventional oxidative stress pathways [22].

Nrf2 has also been shown to modulate tau pathology by diminishing hyperphosphorylated tau levels through stimulation of autophagic degradation. Nrf2 enhances the expression of nuclear dot protein 52 (NDP52), an autophagy-related protein that facilitates the clearance of hyperphosphorylated tau via the autophagic-lysosomal pathway [38,39]. Nrf2 deficiency led to reductions in autophagy markers p62/SQSTM1 (1.9-fold) and NDP52 (7.8-fold), highlighting the critical role of Nrf2 in regulating tau degradation (Fig. 1) [40]. Consistent with these findings in Nrf2 knockout mice, phosphorylated tau levels were significantly elevated, with 2.9- and 2.7-fold increases observed at Ser262/Ser356 (12E8) and Ser396/Ser404 (PHF1) epitopes, respectively, compared to wild-type mice. Furthermore, total tau levels showed a 1.8- to 2.1-fold increase [38]. Treatment with sulforaphane (SFN), a known Nrf2 activator, reduced phosphorylated tau levels by 30 ​% and 70 ​% in primary cortical neurons and CN1.4 mouse cortical cells, respectively, at Ser262/Ser356, by 50 ​% and 43 ​% at Ser396/Ser404. Significantly, SFN treatment did not affect neuronal viability, confirming that tau reduction was not due to cytotoxicity [40]. These findings suggest that pharmacologic activation of Nrf2 could be a promising therapeutic strategy to delay the onset of AD, slow disease progression by targeting tau pathology through enhanced autophagic clearance mechanisms, and attenuate cognitive dysfunction [41]. However, despite the promising therapeutic effects of sulforaphane in preclinical models of AD and related neurodegenerative disorders [41], its electrophilic properties cause off-target side effects [42]. Nrf2 also regulates the expression of lysosomal-associated membrane protein 2A (LAMP2A), a key mediator of chaperone-mediated autophagy, broadly linking Nrf2 to dysregulated proteostasis in AD [39,43].

In addition to autophagy, other mechanisms regulate tau pathology, such as activating the regulatory protein peptidylprolyl *cis*-trans isomerase (Pin1). Pin1 is pivotal in modulating tau phosphorylation by acting as a molecular switch that maintains proper tau function and structural stability [44]. Pin1 catalyzes the *cis*-trans isomerization of specific phosphorylated serine/threonine-proline motifs, a process crucial for modulating tau's interaction with kinases and phosphatases. Notably, Pin1 also regulates glycogen synthase kinase-3 beta (GSK-3β) and protein phosphatase 2A (PP2A), key enzymes that control tau phosphorylation and dephosphorylation, respectively. Both reduced levels and oxidative modification of Pin1 have been observed in both AD and mild cognitive impairment (MCI) brains [44]. These alterations impair Pin1's ability to restore tau's healthy physiological state, contributing to pathological hyperphosphorylation and subsequent tau aggregation. Additionally, GSK3β-mediated hyperphosphorylation of tau causes it to aggregate into neurofibrillary tangles, a hallmark of AD. This hyperphosphorylation destabilizes microtubules, disrupting neuronal transport and contributing to neurodegeneration [45]. GSK3β also phosphorylates Nrf2 at specific serine residues in the Neh6 domain, creating a recognition motif for the β-TrCP (β-Transducin repeat-containing proteins) E3 ubiquitin ligase adapter protein. This phosphorylation targets Nrf2 for ubiquitination and subsequent degradation by the proteasome. While this typically helps maintain cellular homeostasis, excessive GSK3β activity in AD can reduce Nrf2 levels and impair the cell's redox homeostasis [46].

Another promising avenue of therapeutic interest is the interplay between Nrf2 and neuroinflammation through its interaction with the chemokine fractalkine (CX3CL1). CX3CL1 activates Nrf2 via the PI3K/AKT signaling pathway to enhance the expression of Nrf2 target genes such as HO-1, which protects against neuroinflammation and oxidative stress. In accordance with these observations, mouse models deficient in Nrf2 or the receptor CX3CR1 exhibit increased microglial and astroglial activation, suggesting a pivotal role for the CX3CL1/Nrf2 axis in modulating the neuroinflammatory response in AD [47]. These findings underscore the potential of targeting the CX3CL1/Nrf2 pathway to mitigate inflammation and enhance neuroprotection in AD (Fig. 1) [39].

Biliverdin reductase-A (BVR-A) is an enzyme that converts biliverdin to bilirubin [48]. Although BVR-A counteracts oxidative stress, it is particularly vulnerable to oxidative damage. It is post-translationally modified by 3-NT and 4-HNE, a feature observed in AD and mild cognitive impairment brains, compromising its functional role [49]. Additionally, BVR-A interacts with insulin receptor substrate-1 (IRS-1), a key regulator of insulin signaling that directs signals from the insulin receptor to the PI3K/Akt or MAPK pathways [50]. BVR-A phosphorylates and inhibits the insulin receptor substrate (IRS) to prevent the aberrant activation of IRS as part of a feedback regulatory loop, with the loss of BVR-A promoting brain insulin resistance [51]. Furthermore, BVR-A participates in a BVR-A/GSK-3β/PPARα axis that regulates hepatic lipid metabolism [52]. Thus, the loss of BVR-A results in increased activity of GSK-3β, increased tau phosphorylation, and mitochondrial dysfunction in AD [53].

Dysregulation of the PI3K/Akt pathway occurs at multiple levels in AD and extends beyond Nrf2 signaling, as oligomeric amyloid-beta peptides can activate PI3K/Akt and the mTORC1 pathway. This triggers a cascade of phosphorylation events, including serine-307 phosphorylation on IRS-1, leading to insulin resistance—a hallmark of AD-associated metabolic dysfunction [49,54,55]. Insulin resistance contributes significantly to impaired glucose metabolism in AD and MCI brains, highlighting the complex interplay between metabolic and signaling pathways in AD pathology.

**Fig. 2:**
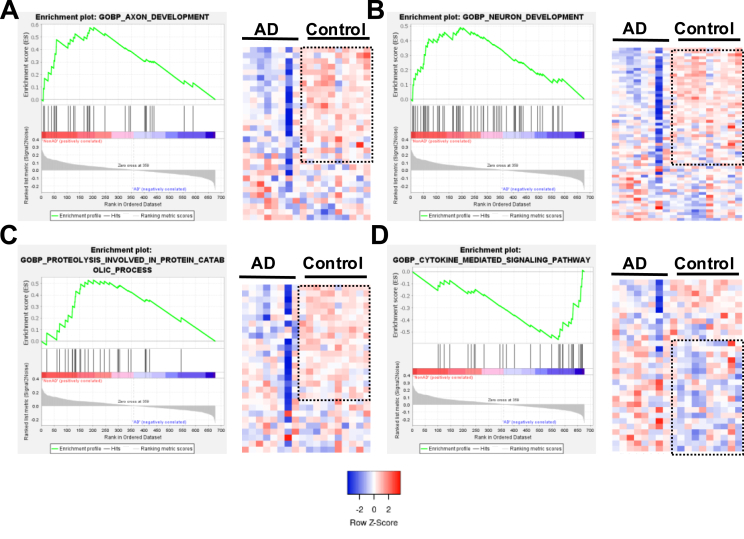
**Nrf2-bound genes regulate neuroprotective pathways in human Alzheimer's Disease.** GSEA analysis of Nrf2 bound loci in human postmortem AD and control hippocampi (GSE36980; AD ​= ​8, Control ​= ​10) showing (A) axon development, (B) neuron development, (C) proteolysis involved in protein catabolic process, (D) cytokine signaling pathways. The dotted rectangle in control represents the driver genes differentially regulated between control and AD data. The gene names and expression values are provided in Table S1.

To further characterize signaling mediated by Nrf2 more broadly, we generated a cumulative Nrf2 binding signature, utilizing the chromatin immunoprecipitation sequencing (ChIP seq) dataset generated by Hokama et al., [56]. This Nrf2-bound gene signature was used to extract the expression data from the postmortem human AD and control hippocampi (GSE36980) [56]. Gene set enrichment analysis indicated that Nrf2-bound genes associated with neuroprotective pathways, such as axon development and neuron development (with significant overlap with genes involved in synaptic plasticity), as well as autophagy (proteolysis involved in protein catabolic process) genes that were significantly downregulated in AD hippocampi compared to controls (Fig. 2A–C) and (Table S1). Significantly, the Nrf2-bound genes involved in cytokine signaling (proinflammatory genes) (Table S1) were upregulated in AD hippocampi compared to controls (Fig. 2D), underscoring the neuroprotective role of Nrf2-mediated signaling in AD pathophysiology.

## The Nrf2-Bach1 signaling axis as a multi-pronged therapeutic strategy for Alzheimer's disease

Despite the stabilization/overexpression of Nrf2 under chronic oxidative stress and inflammation, its ability to drive the expression of antioxidant genes is frequently inadequate in neurodegenerative conditions. This is partly due to feedback mechanisms, with prolonged activation of Nrf2 leading to an upregulation of transcriptional repressors like Bach1 [57]. It has been shown that silencing Bach1 in cells from older adults increases the expression of Nrf2-regulated genes, indicating a role for Bach1 as a repressor of Nrf2 signaling [58]. Consequently, silencing/inhibiting Bach1 could restore Nrf2 functionality and reinstate antioxidant defenses in aging and neurodegenerative diseases [24]. Multiple studies have further confirmed that Bach1 negatively regulates Nrf2 signaling to inhibit its activity [59,60]. For example, in individuals with Down syndrome (trisomy 21), elevated Bach1 expression, arising due to its genetic location on chromosome 21, suppresses Nrf2 target genes, thereby increasing susceptibility to AD-like dementia [60]. Although studies investigating Bach1 alterations in AD are limited, emerging evidence suggests that alterations in Bach1 activity occur in the AD brain [61].

Several recent studies have also reported the beneficial effects of enhancing Nrf2 signaling cascades, which range from targeting either Nrf2 alone or inhibiting Bach1 in combination with Nrf2 activation. Nrf2 activators span a range of compounds, including natural phytochemicals, cannabinoids, and synthetic agents like dimethyl fumarate (DMF), a drug with the most extensive clinical use [62]. DMF promotes Nrf2 activation in the cytoplasm and facilitates Bach1's exit from the nucleus, thereby relieving Nrf2 repression [63]. In SH-SY5Y cells exposed to Aβ1-42 or 6-hydroxydopamine, DMF increased cell viability and reduced intracellular reactive oxygen species (ROS), likely by activating HO-1 [24]. Recently, the benzimidazole derivative *N*-(2-(2-hydroxyethoxy)ethyl)-1-methyl-2-((6-(trifluoromethyl)benzo[*d*]thiazol-2-yl)amino)-1*H*-benzo[*d*]imidazole-5-carboxamide, designated as HPPE, was discovered with the ability to both inhibit Bach1 and directly activate Nrf2 simultaneously [64]. This novel compound is considered superior to canonical Nrf2 activators, which are electrophilic agents that target KEAP1 thiols to liberate Nrf2 but also result in nonspecific modification of active cysteine residues in other cellular proteins. Treatment with HPPE has shown significant reductions in oxidative stress markers, glial activation, neuroinflammation, and enhanced expression of neuroprotective genes, affirming its neuroprotective effects [64]. Contrary to the prevailing view that Bach1 is a repressor of Nrf2 and inhibits Nrf2 target genes, analysis of Bach1-bound genomic loci revealed signatures of both Nrf2-dependent and independent genes [65,66]. Given that ARE motifs are similar to activator protein-1 (AP-1) motifs, Bach1 may regulate non-Nrf2 target genes by associating with other transcription factors, such as the (E-twenty-six) ETS family [65]. These observations support the view that Bach1 inhibition can upregulate Nrf2-dependent and Nrf2-independent neuroprotective genes. Thus, unlike DMF, which activates Nrf2 through its electrophilic properties and results in off-target effects, the bipartite small molecule HPPE is a non-electrophile and represents a promising therapeutic agent for AD [64] that simultaneously activates Nrf2 and inhibits Bach1 to upregulate both Nrf2-dependent and Nrf2-independent genes.

## Current landscape of Alzheimer's disease drug development and clinical trials

The commonly used cholinesterase inhibitors such as Donepezil, Rivastigmine, and Galantamine aim to improve cognitive symptoms by enhancing cholinergic neurotransmission by inhibiting acetylcholinesterase, which breaks down acetylcholine. However, while these drugs may provide brief symptomatic relief, they do not slow or halt disease progression. Additionally, their benefits diminish over time as they do not address the underlying pathological processes [[67], [68], [69], [70]].

Over the past few decades, research into AD drug development has predominantly centered around its two major pathological hallmarks: Aβ plaques and tau NFTs. These targets have formed the backbone of most clinical trials, with amyloid clearance therapies recently taking the spotlight. Monoclonal antibodies, such as Aducanumab, Lecanemab, Donanemab, and Gantenerumab, have been developed to bind and remove Aβ deposits in the brain [[71], [72], [73], [74], [75]]. Despite early signs of amyloid reduction in clinical trials, the benefits have been modest, with improvements in cognitive decline either marginal or clinically insignificant. Thus, while these recently approved amyloid-targeting therapies have generated excitement, their overall efficacy remains under debate due to mixed results and concerns about side effects, such as cerebral edema and hemorrhage [72,[76], [77], [78], [79]]. In parallel, tau-targeting therapies have also entered the clinical trial arena to prevent tau aggregation or stabilize microtubules. Tau immunotherapies, including Semorinemab, Tilavonemab (ABBV-8E12), and Zagotenemab, are currently being investigated for their potential to reduce neurofibrillary tangles and alleviate cognitive symptoms [[80], [81], [82], [83]]. While initial trials have shown some promise, tau-based interventions have yet to successfully target intracellular tau deposits to halt or reverse disease progression [84].

Beyond amyloid and tau, efforts are expanding to include anti-inflammatory strategies and metabolic modulators [[85], [86], [87]]. Another novel approach that has recently emerged in preclinical AD studies is the augmentation of persulfidation to stabilize cellular signaling and protect reactive cysteine residues [48,88,89]. Furthermore, efforts to modulate neurotransmitter systems - such as the cholinergic and glutamatergic pathways-continue to evolve, with drugs like cholinesterase inhibitors and NMDA receptor antagonists being part of the symptomatic treatment toolbox [[90], [91], [92]]. Trials involving therapies like XPro1595, which targets TNF-alpha, and Azeliragon, a RAGE inhibitor, have yielded mixed results in addressing inflammation. It is particularly challenging to strike the right balance between suppressing harmful neuroinflammation and maintaining protective immune responses, as overly dampening the immune system can hinder its beneficial roles [93,94]. Despite these extensive efforts, AD clinical trials face the overwhelming obstacle that most interventions yield only incremental improvements at best.

Several promising therapeutic candidates, such as Lanabecestat, a BACE1 inhibitor essential for Aβ peptide production, along with Crenezumab and Solanezumab, monoclonal antibodies designed to target different Aβ aggregates, have failed to demonstrate efficacy in clinical trials [95,96]. Other BACE inhibitors, like Verubecestat and Elenbecestat, failed in clinical trials due to limited efficacy and notable adverse effects, including cognitive decline [95,[97], [98], [99]]. This failure is partly attributed to the fact that BACE1 plays a role in vital synaptic functions beyond amyloid production, meaning that inhibiting BACE1 can disrupt critical biological processes. A key challenge remains in finding a balance between reducing amyloid levels and preserving the physiological functions of BACE1. These failures have prompted the exploration of alternative therapeutic approaches that move beyond the inhibition of protein aggregation, with particular attention turning to oxidative stress as a critical pathological driver in AD.

## Shortcomings of the existing therapeutic strategies

A significant limitation of current clinical trials in AD lies in their narrow focus on the hallmark pathological features, such as amyloid and tau, without consideration of interconnected biological processes that drive AD progression. Targeting amyloid without addressing the accompanying oxidative stress, synaptic loss, or immune dysfunction may explain the lack of substantial cognitive improvements in clinical trials. Moreover, amyloid and tau therapies come with their own set of challenges. For example, safety concerns often overshadow the efficacy of amyloid-targeting treatments, as patients receiving these therapies frequently experience adverse events like amyloid-related imaging abnormalities (ARIA), leading to cerebral swelling and microbleeds [100,101]. While still under investigation, tau-targeting therapies have also struggled to demonstrate robust, consistent benefits in reversing cognitive decline in AD [102,103].

Small amyloid-beta (Aβ) oligomers (AβOs) are critical contributors to AD pathogenesis, exerting profound effects on oxidative stress, mitochondrial dysfunction, and synaptic plasticity [104]. These oligomers are recognized as significant therapeutic targets due to their neurotoxic and synaptotoxic effects, which exacerbate synaptic dysfunction and neuroinflammation. Lipid peroxidation is a critical consequence of AβOs accumulation [[105], [106], [107]], which leads to extensive cellular damage by forming reactive aldehydes such as 4-HNE and malondialdehyde (MDA). These electrophilic aldehydes amplify neuronal injury by forming adducts with proteins, nucleic acids, and membrane lipids, exacerbating neurodegeneration. Mechanistically, AβOs disrupt membrane homeostasis by inducing polyunsaturated fatty acids (PUFAs) peroxidation, such as arachidonic acid and docosahexaenoic acid (DHA), which are abundant in neuronal membranes. The oxidation of PUFAs generates lipid peroxides that decompose into reactive aldehydes, further impairing membrane fluidity, synaptic function, and intracellular signaling cascades [108,109]. This lipid peroxidation-driven toxicity is a major contributor to neuronal dysfunction in AD.

Therapeutic approaches, including small molecules and antibodies, aimed at counteracting the toxic effects of Aβ oligomers, thereby protecting synapses and improving cognitive deficits, as observed in AD models [110,111], are also currently insufficient. Notably, Sigma-2/PGRMC1 receptors, which mediate the binding of Aβ oligomers to neurons, have also emerged as promising targets for small-molecule therapeutics [112]. Despite their potential, developing specific and effective therapies remains challenging due to the transient and structurally heterogeneous nature of Aβ oligomers. Furthermore, it is critical to ensure that such treatments do not interfere with the normal physiological functions of Aβ, necessitating a balanced and precise therapeutic strategy.

Another critical area for improvement is the timing of the intervention. Many clinical trials enroll subjects in the later stages of AD, at which point significant neuronal loss and cognitive impairment have already occurred [113,114]. By this time, removing amyloid or reducing tau may not be sufficient to reverse neuronal damage and cognitive recovery, suggesting that earlier intervention, perhaps even before the emergence of clinical symptoms, may be necessary. However, this shifts the challenge to early diagnosis, which is still fraught with difficulties, as reliable biomarkers for detecting preclinical AD in people are limited.

Lastly, AD is a highly heterogeneous disease influenced by genetic, environmental, and lifestyle factors [115]. This variability means that a one-size-fits-all approach is unlikely to be effective. Precision medicine, tailoring treatments to the individual's unique genetic or biomarker profile, has yet to gain widespread adoption in AD trials, limiting the potential for targeted interventions. The collective shortcomings of current clinical trials highlight the need for innovative strategies that address the multifactorial nature of AD. This has paved the way for exploring pathways beyond amyloid and tau, such as oxidative stress regulation, neuroinflammation, and other neuroprotective pathways as potential therapeutic targets.

## Future perspectives

AD is a profoundly complex and multifaceted disorder in which diverse pathological processes converge to drive neurodegeneration. From protein misfolding and oxidative stress to chronic inflammation and metabolic disruption, the complexity of AD challenges our understanding and complicates efforts to develop effective treatments. Thus, singular approaches targeting only the classic amyloid and tau proteinopathies are insufficient for comprehensive treatment. Thus, identifying and modulating multifaceted therapeutic targets is paramount for advancing neurotherapeutic interventions in AD.

The Nrf2/Bach1 signaling axis offers a promising strategy to simultaneously address multiple pathological processes in AD. While preclinical studies show promise, challenges remain in translating Nrf2-targeted therapies to clinical practice, particularly with current strategies utilizing electrophilic pharmacophores (e.g., Tecfidera and Omaveloxolone) that cause side effects by broadly affecting proteins besides KEAP1 [42,116]. A safer approach to activate Nrf2 is to use non-electrophilic displacement activators targeting the KEAP1 Kelch domain, thus dissociating Nrf2 from KEAP1 [15,116]. Moreover, Nrf2 stabilization and its subsequent activation induce the expression of the transcriptional repressors of Nrf2 via a negative feedback mechanism [15,66,116]. Therefore, interventions that do not prevent this feedback loop are insufficient to combat disease pathogenesis. The critical balance between Nrf2 activation and Bach1 repression suggests that simultaneous activation of Nrf2 and inhibition of Bach1 is necessary to achieve the maximal therapeutic benefit. This is because Bach1 is one of the gene targets for Nrf2, and thus, Nrf2 activation without Bach1 inhibition can only exert short-term benefits due to the negative feedback regulatory mechanism [117,118]. Bipartite pharmacological agents, such as HPPE that activate Nrf2 while suppressing Bach1, could optimize the antioxidant response, reduce oxidative stress, and promote neuronal resilience. Such interventions may also mitigate other vital pathological processes in AD, including impaired proteostasis and heightened inflammation, offering a comprehensive, multipronged therapeutic approach. As research advances, the challenge remains in identifying potent and specific modulators of this pathway that can cross the blood-brain barrier and exert long-term therapeutic benefits without adverse effects.

## Author contributions

P·S., S.M.S., A.A.P., B.D.P., and B.T. conceived and wrote the manuscript. P.S. and S.M.S. created figures. P.S., S.M.S., A.A.P., B.D.P., and B.T. edited and reviewed the manuscript. All authors have read and agreed to the published version of the manuscript.

## Declaration of competing interest

The authors declare that they have no known competing financial interests or personal relationships that could have appeared to influence the work reported in this paper.

## Abbreviations

3-NT: 3-nitrotyrosine
4-HNE: 4-hydroxynonenal
Aβ: Amyloid beta
AβOs: Amyloid-beta oligomers
AD: Alzheimer's Disease
AP-1: Activator Protein-1
APOE: Apolipoprotein E
AREs: Antioxidant response elements
ARIA: Amyloid-related imaging abnormalities
BACE-1: Beta-site amyloid precursor protein cleaving enzyme 1
Bach1: BTB and CNC homology 1
BVR-A: Biliverdin reductase-A
ChIP: Chromatin immunoprecipitation sequencing
CUL3: Cullin 3
CX3CL1: Chemokine fractalkine
DHA: Docosahexaenoic acid
DMF: Dimethyl fumarate
ER: endoplasmic reticulum
ETS: E-twenty-six
GCL: Glutamate-Cysteine Ligase
GPX4: Glutathione peroxidase
GSK-3β: Glycogen synthase kinase-3 beta
HO1: Heme oxygenase-1
HPPE: *N*-(2-(2-hydroxyethoxy)ethyl)-1-methyl-2-((6-(trifluoromethyl)benzo[*d*]thiazol-2-yl)amino)-1*H*-benzo[*d*]imidazole-5-carboxamide
IRS-1: Insulin receptor substrate-1
KEAP1: Kelch-like ECH-associated protein 1
LAMP2: Lysosomal-associated membrane protein 2A
MAF: Musculoaponeurotic fibrosarcoma
MCI: Mild cognitive impairment
MDA: Malondialdehyde
NDP52: nuclear dot protein 52
NFTs: Neurofibrillary tangles
NOX4: NADPH oxidase 4
Nrf2: Nuclear factor E2-related factor 2
OS: Oxidative stress
Pin1: Peptidylprolyl *cis*-trans isomerase
PGRMC1: Progesterone Receptor Membrane Component 1
PP2A: Protein phosphatase 2A
PPARα: Peroxisome proliferator-activated receptor alpha
PRDX6: Peroxiredoxin 6
PUFA: Polyunsaturated fatty acids
RBX1: RING Box Protein 1
ROS: Reactive oxygen species
SFN: Sulforaphane
SOD: Superoxide dismutase
TXNRD1: Thioredoxin reductase 1
Ub: Ubiquitin
WT: Wild type
xCT: cystine transporter
β-TrCP: beta-Transducin repeat-containing protein
4-HNE: 4-hydroxy 2-nonenal

## Appendix A Supplementary data

Multimedia component 1 Pathways and respective Nrf2-bound gene signatures in the pathophysiology of human Alzheimer's Disease. Multimedia component 1
